# Design of Acceptors with Suitable Frontier Molecular Orbitals to Match Donors via Substitutions on Perylene Diimide for Organic Solar Cells

**DOI:** 10.3390/ijms17050721

**Published:** 2016-05-13

**Authors:** Xiaoli Lv, Zhuoxin Li, Songyang Li, Guoyou Luan, Dadong Liang, Shanshan Tang, Ruifa Jin

**Affiliations:** 1 College of Resource and Environmental Science, Jilin Agricultural University, Changchun 130118, China; lvxiaoli66@126.com (X.L.); lizhuoxin6706@sina.com (Z.L.); lsy3153204@126.com (S.L.); dadongliang@126.com (D.L.); 2 College of Chemistry and Chemical Engineering, Chifeng University, Chifeng 024000, China; ruifajin@163.com

**Keywords:** perylene diimide derivatives, frontier molecular orbitals, optical properties, charge transport property, organic solar cells

## Abstract

A series of perylene diimide (PDI) derivatives have been investigated at the CAM-B3LYP/6-31G(d) and the TD-B3LYP/6-31+G(d,p) levels to design solar cell acceptors with high performance in areas such as suitable frontier molecular orbital (FMO) energies to match oligo(thienylenevinylene) derivatives and improved charge transfer properties. The calculated results reveal that the substituents slightly affect the distribution patterns of FMOs for **PDI-BI**. The electron withdrawing group substituents decrease the FMO energies of **PDI-BI**, and the electron donating group substituents slightly affect the FMO energies of **PDI-BI**. The di-electron withdrawing group substituents can tune the FMOs of **PDI-BI** to be more suitable for the oligo(thienylenevinylene) derivatives. The electron withdrawing group substituents result in red shifts of absorption spectra and electron donating group substituents result in blue shifts for **PDI-BI**. The –CN substituent can improve the electron transport properties of **PDI-BI**. The –CH_3_ group in different positions slightly affects the electron transport properties of **PDI-BI**.

## 1. Introduction

Organic solar cells (OSCs) with high power conversion efficiencies (PCEs) exceeding 10% have been fabricated [1]. Among them, organic small molecules as solar cell materials based on π-conjugate polymers are attractive because of their rapid energy payback time [2], low cost, flexibility, light weight, solution-based processing, and the capability to fabricate flexible large-area devices [3]. The PCEs of the OSCs have exceeded 11% when the conventional fullerene as the acceptors [4,5]. However, the fullerene and its derivative acceptors have several limitations, such as costly production, fixed band alignment, and limited optical absorption, which significantly prevent the development of new donor materials. Thus, developing and investigating novel acceptors has become a focus around the world. Up to now, many small molecule acceptors have been reported, such as 9,9′-bifluorenylidene [6,7], dicyan substituted quinacridone [8], diketopyrrolopyrrole derivatives [9,10], vinazene [11,12], fluoranthene-fused imide [13,14], naphthalene diimides [15,16], electron-deficient pentacenes [17], and perylene diimides (PDIs) [18,19,20,21]. Among the small molecule acceptors, PDI and its derivatives have attracted much attention in the past decade due to their superior optical and electric properties—for example, excellent chemical, photochemical, and thermal stabilities [22], high absorption (450 and 650 nm) [23], promising electron mobility [24,25,26], and excellent electron affinity [27]. Yao *et al.* obtained solar cells with 4.34% efficiency on the basis of PDI [21]. Nguyen *et al.* prepared the PDI bulk heterojunction solar cell [1]. Shin *et al.* obtained OSCs with a power conversion efficiency of 0.18% under AM 1.5 using PDI derivatives as acceptors [28]. Zhang *et al.* [29,30] and Tang *et al.* [31] deigned a series of PDI derivatives and calculated their properties.

**Figure 1:**
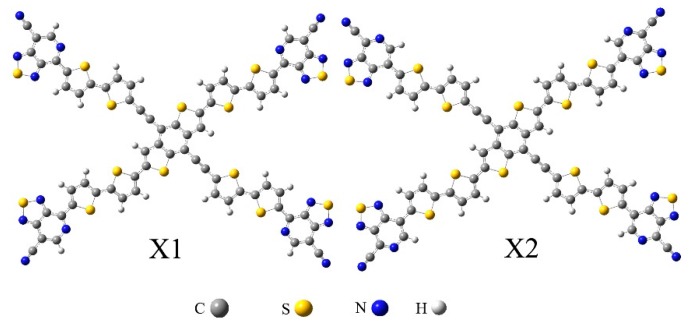
The structures of donors **X1** and **X2** from Ref. [32].

Won Suk Shin *et al.* prepared some PDI derivatives, and molecule **PDI-BI** had suitable properties as a solar cell acceptor [28]. In this manuscript, in order to improve the performance of **PDI-BI**, we have designed various **PDI-BI** derivatives (Table 1), which have different functional groups, to find the most promising acceptors with suitable frontier molecular orbital energies (FMOs) to match the OSC donor oligo(thienylenevinylene) derivatives (**X1** and **X2**, Figure 1) with favourable properties designated by Yong *et al.* [32]. Generally, the higher the lowest unoccupied molecular orbital (LUMO) of the acceptor, the larger the open circuit voltage (*V*_oc_), because the difference in energy between the highest occupied molecular orbital (HOMO) energy of the donor and LUMO of the acceptor is in direct proportion to the *V*_oc_. In addition, to ensure separation of charge, the differences between the LUMO energies of the donor and the acceptor should be greater than 0.30 eV [33]. Considering the fact that the substituent groups affect the molecular properties significantly, we designed two kinds of molecules (**PDI-BI-1-26**) to study the push (–CH_3_) and pull (–CN and –NO_2_) substituent groups effects. The density function theory (DFT) [34] has been used for evaluating a variety of ground state properties of these molecules, such as FMO, including HOMO and LUMO energies, and the HOMO–LUMO gaps (*E*_g_). The optical properties (absorption spectra) of the designed molecules have been predicted by the time dependent DFT [35,36,37] approach (TD-DFT). The reorganization energy (λ) was also calculated. Additionally, we discussed the correlation between structures and properties of these molecules.

## 2. Results and Discussion

### 2.1. Frontier Molecular Orbitals

**Figure 2:**
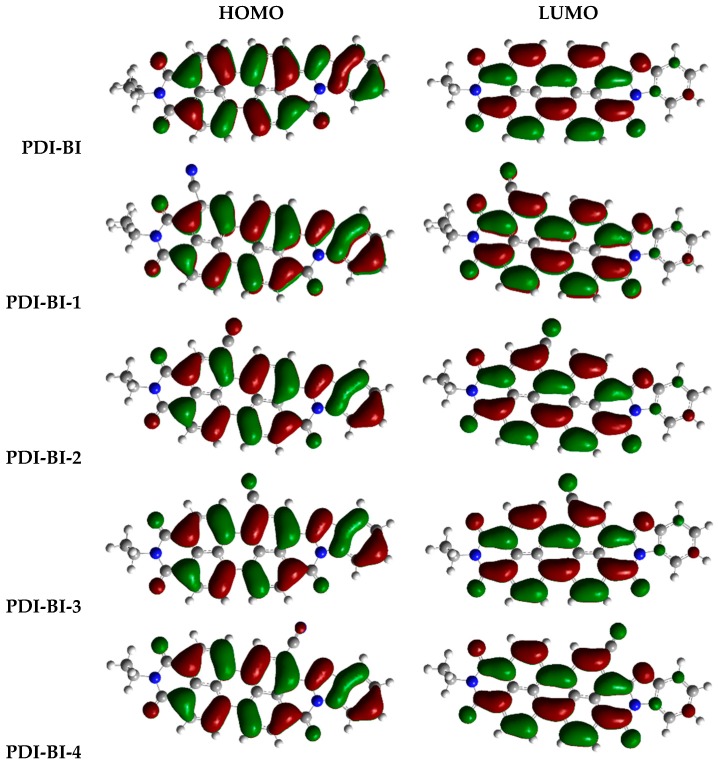

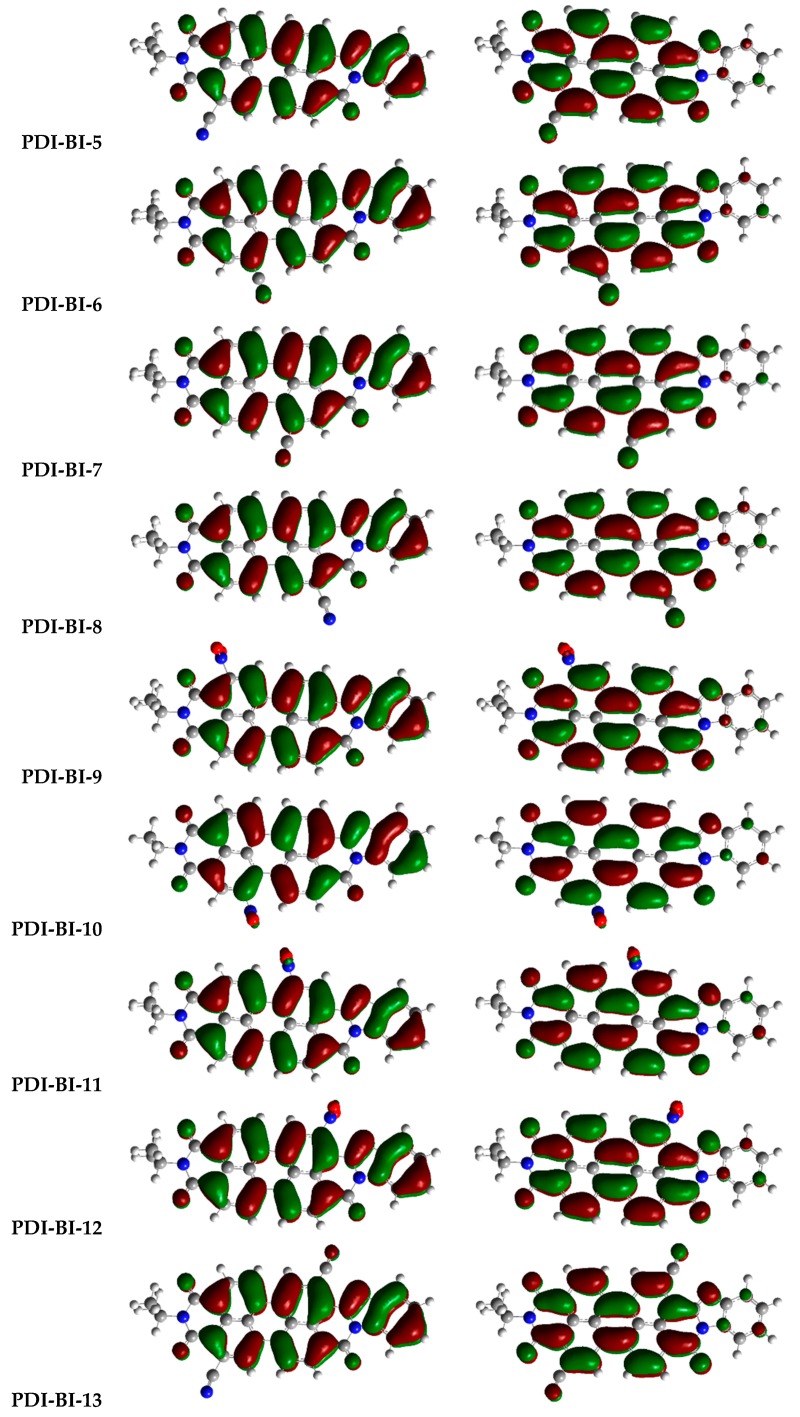

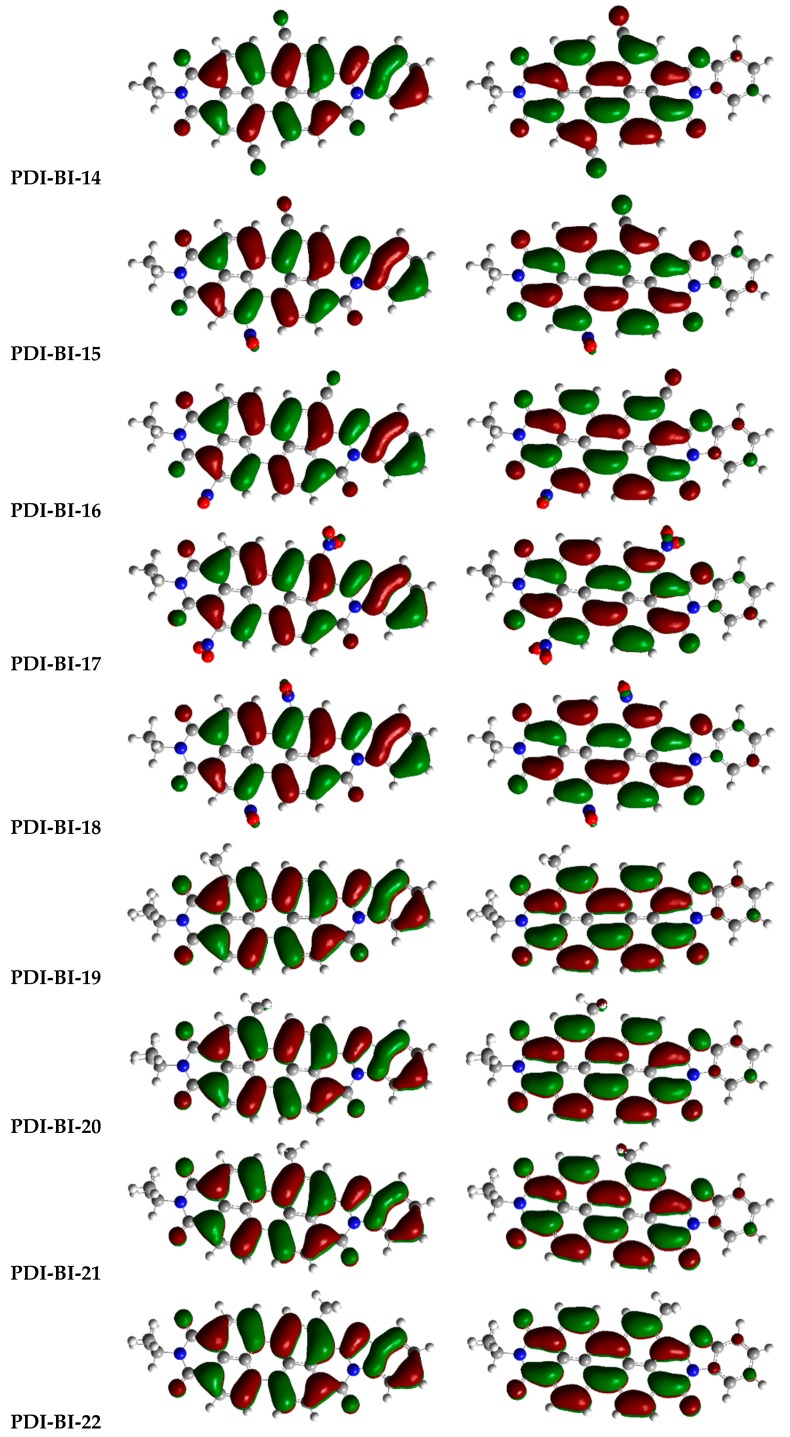

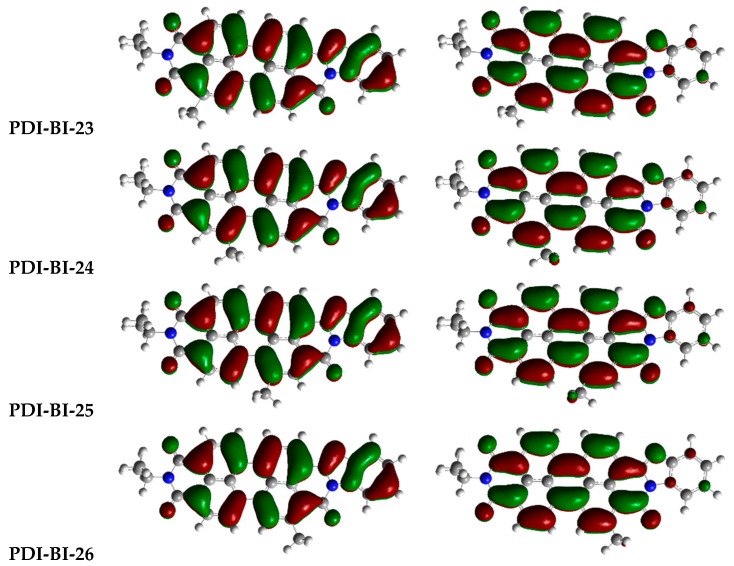
The distribution patterns of FMO for **PDI-BI** and its derivatives at the CAM-B3LYP/6-31G(d) level.

**Figure 3:**
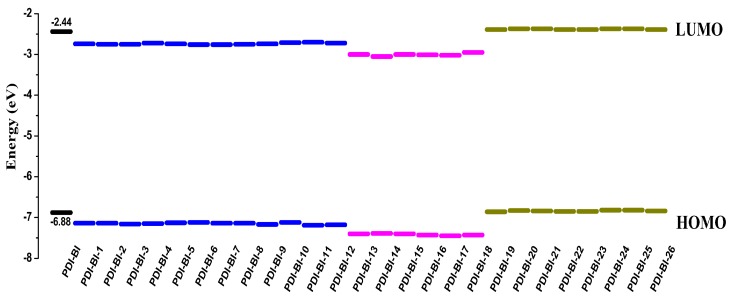
The *E*_HOMO_ and *E*_LOMO_ values of FMO for **PDI-BI** and its derivatives at the CAM-B3LYP/6-31G(d) level. The black is PDI-BI, the blue means mono-pull substituent, the purple represents di-pull substituent, and the olive is mono-push substituent.

The electronic and optical properties of molecules are related to the values of FMOs and *E*_g_. Thus, in order to gain insight into the influence of the optical and electronic properties, the distribution patterns of the FMOs for the designed molecules are studied, and the electronic density contours of the designed molecules in ground states are shown in Figure 2. The evaluations of HOMO and LUMO energies (*E*_HOMO_ and *E*_LOMO_) for designed molecules are plotted in Figure 3 and listed in Table 2.

From Figure 2, one can see that the FMOs are spread over the entire molecule for the designed molecules. This indicates that there is great spatial overlap between the HOMO and LUMO, and the transition from HOMO to LUMO may lead to strong optical adsorption. As shown in Figure 3 and Table 2, the –CN and –NO_2_ groups in different substituent positions can decrease the *E*_HOMO_, *E*_LOMO_, and *E*_g_ values of **PDI-BI**, except that –NO_2_ in 3 or 4-position increases the *E*_g_ value of **PDI-BI** (**PDI-BI-11** and **PDI-BI-12**), and the deviations of *E*_HOMO_, *E*_LOMO_, and *E*_g_ values for molecules **PDI-BI-1-12** are similar, respectivety. For molecules **PDI-BI-1-8**, the decrease of the *E*_HOMO_ value is the largest when the –CN group is in the 3-position of **PDI-BI**. The decrease of the *E*_LOMO_ value is the largest when the –CN group is in the 6 or 7-position of **PDI-BI**. The *E*_g_ value is the smallest when the –CN group is in the 6-position of **PDI-BI**. For molecules **PDI-BI-9-12**, the decrease of the *E*_HOMO_ value is the largest when the –NO_2_ group is in the 3-position of **PDI-BI**. The decrease of the *E*_LOMO_ value is the largest when the –NO_2_ group is in the 1-position of **PDI-BI**. The *E*_g_ value is the smallest when the –NO_2_ group is in the 2-position of **PDI-BI**. The di-CN, di-NO_2_, or –CN and –NO_2_ groups in different substituent positions can decrease the *E*_HOMO_, *E*_LOMO_, and *E*_g_ values of **PDI-BI**, except that the –NO_2_ in 3 and 6-positions increase the *E*_g_ values of **PDI-BI** (**PDI-BI-18**), and the decreased amounts of *E*_HOMO_, *E*_LOMO_, and *E*_g_ values for molecules **PDI-BI-13-18** are similar, respectively. The *E*_HOMO_ value decrease is the largest when the –NO_2_ groups are in the 4 and 5-positions of **PDI-BI**. The decrease of the *E*_LOMO_ value is the largest when the –CN groups are in the 3 and 6-positions of the molecule **PDI-BI**. The *E*_g_ value is the largest when the –NO_2_ groups are in the 3 and 6-positions the molecule **PDI-BI**. For molecules **PDI-BI-19-26**, the –CH_3_ group in different substituent position affects the *E*_HOMO_, *E*_LOMO_, and *E*_g_ of **PDI-BI** slightly. These results reveal that the electron withdrawing substituents can decrease the *E*_HOMO_, *E*_LOMO_, and *E*_g_ values of **PDI-BI**. The electron donating substituents affect *E*_HOMO_, *E*_LOMO_, and *E*_g_ values of **PDI-BI** slightly.

**Figure 4:**
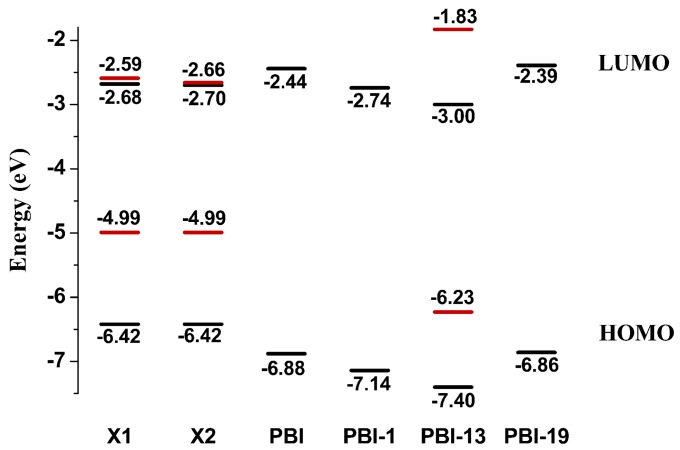
Evaluation of the computed HOMO and LUMO energies for **PDI-BI**, **PDI-BI-1**, **PDI-BI-13**, and **PDI-BI-19** as well as the HOMO and LUMO energies for **X1** and **X2** at the CAM-B3LYP6-31G(d)//PBE0/6-31G(d) level. The black line represents singlets, and the red line represents triplets.

The *E*_HOMO_ and *E*_LOMO_ values of FMO for molecules **X1**, **X2**, **PDI-BI-1**, **PDI-BI-13**, and **PDI-BI-19** are plotted in Figure 4. The molecules **PDI-BI-1**, **PDI-BI-13**, and **PDI-BI-19** are the representatives of the different kinds of substituent molecules, respectively. As shown in Figure 4, one can see that the LUMO energies of **PDI-BI-13** are lower (0.32 and 0.30 eV) than those of **X1** and **X2**, which indicates that **PDI-BI-13** is suitable for the FMOs of **X1** and **X2**, respectively. That is to say, molecules **PDI-BI-14**, **PDI-BI-15**, **PDI-BI-16**, and **PDI-BI-17** are also suitable for the FMOs of **X1** and **X2**, respectively. This reveals that the di-CN, di-NO_2_, or –CN and –NO_2_ groups substituents can decrease the FMOs of **PDI-BI.** Thus, proper substitutions can tune the FMOs of **PDI-BI** to be more suitable to **X1** and **X2**. Moreover, we calculated the triplet energies of **X1**, **X2**, and **PDI-BI-13**. The calculated results show that the triplet energies are higher than the corresponding singlet energies for **X1**, **X2**, and **PDI-BI-13**, respectively. This indicates that there may be no triplet loss when **X1**, **X2**, and **PDI-BI-13** are used as the candidates for OSCs devices [38,39,40].

### 2.2. Absorption Spectra

**Figure 5:**
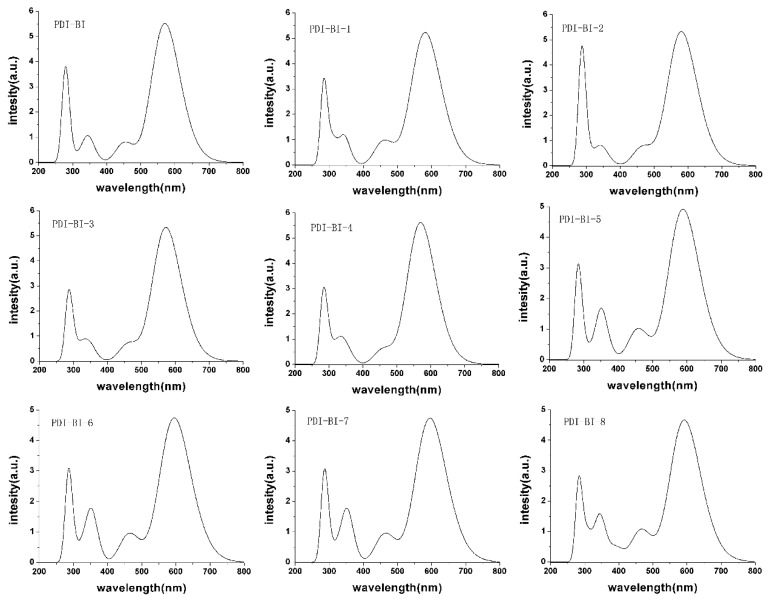

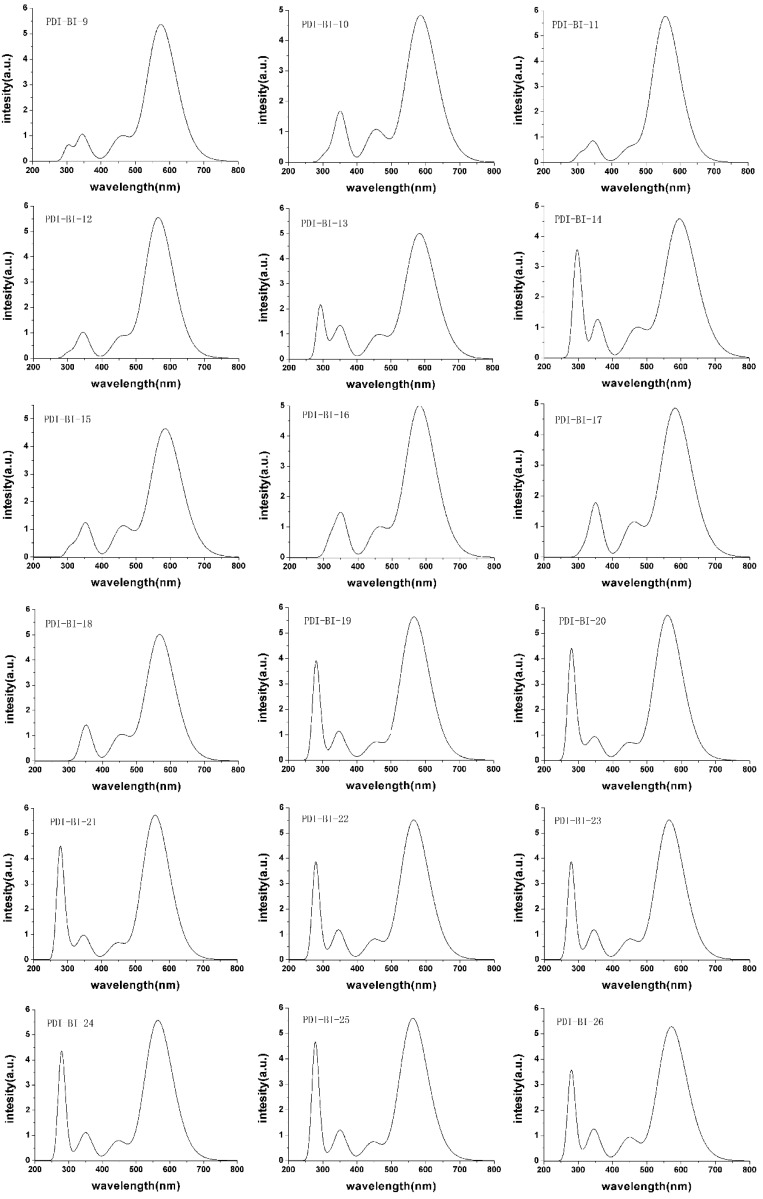
The calculated absorption spectra of **PDI-BI** and its derivatives (value of full width at half maximum is 3000 cm^−1^).

The longest and the shortest wavelengths of the absorption spectra (λ_max_ and λ_min_) and adsorption region (*R*) of the designed molecules are listed in Table 2. The simulated adsorption spectra, plotted using GaussSum 1.0 [41], are shown in Figure 5. The first 20 excited states were considered.

As shown in Table 2 and Figure 5, the –CN group in different positions could increase the λ_abs-max_ and λ_abs-min_ values of **PDI-BI**, respectively, except the –CN group in 4-position could decrease the *λ*_abs-max_ value of **PDI-BI** slightly. The –CN group in the 5, 6, 7, or 8-position can increase the *R* values of **PDI-BI**, and the *R* value increase is larger than the other positions when the –CN group in the 6-position. For –NO_2_ substituent molecules, the λ_abs-max_ values are, in increasing order, **PDI-BI-11** < **PDI-BI-12** < **PDI-BI** < **PDI-BI-9** < **PDI-BI-10**, the λ_abs-min_ values are, in decreasing order, **PDI-BI-10** > **PDI-BI-11** ≈ **PDI-BI-12** > **PDI-BI-9** > **PDI-BI**, and the *R* values are in the order **PDI-BI-11** < **PDI-BI-12** < **PDI-BI-9** < **PDI-BI-10** < **PDI-BI**. This shows that the –NO_2_ group in 2-position could produce a larger increase of λ_abs-max_ and λ_abs-min_ values than the other positions for **PDI-BI**, and the –NO_2_ group in 3-position could produce a larger decrease of the *R* value than the other positions for **PDI-BI**. For di-substituent molecules, the substituent groups could increase the λ_abs-max_ and λ_abs-min_ values of **PDI-BI**, respectively, except the di-NO_2_ groups in 3 and 6-position decrease the λ_abs-max_ value of **PDI-BI,** obviously. The di-substituents could decrease the *R* values of **PDI-BI**, respectively, except the di-CN groups in 3 and 6-position increase the *R* value of **PDI-BI** significantly. The –CH_3_ groups in different positions affect the λ_abs-max_, λ_abs-min_, and *R* values of **PDI-BI** slightly. These results reveal that the mono-pull group can increase the λ_abs-max_, λ_abs-min_, and *R* values of **PDI-BI**, and the push group affects the λ_abs-max_, λ_abs-min_, and *R* values of **PDI-BI** slightly. Among these molecules, **PDI-BI-14** has the largest λ_abs-max_ value and **PDI-BI-6** has the largest *R* value, which indicates that it could be a good candidate for the solar cell acceptor.

### 2.3. Reorganization Energy

The charge transport property of material is important to design the acceptor for a solar cell device, and the reorganization energy plays a role in charge transport and charge separation. It is well-known that the lower the λ values, the better the charge transport property. Thus, we calculated the λ_e_ and λ_h_ values of **PDI-BI** and its derivatives. The calculated results are listed in Table 3.

As shown in Table 3, the –CN group in different positions can decrease the λ_e_ values and increase the λ_h_ values of **PDI-BI**. This implies that the –CN substituent can improve the electron transport property of **PDI-BI**. The –CN substituent in the 4-position (**PDI-BI-4**) owns the largest electron transfer rate. For the –NO_2_ substituent molecules, the substituent groups can increase the λ_e_ and λ_h_ values of **PDI-BI**, except the –NO_2_ group in 1 or 3 position, which can decrease the λ_e_ values of **PDI-BI** slightly. For the di-substituent molecules, the substituent groups can increase the λ_e_ and λ_h_ values of **PDI-BI**, except the di-CN groups (**PDI-BI-13** and **PDI-BI-14**) and –CN in 4-position and –NO_2_ in 5-position (**PDI-BI-16**) substituents, which can decrease the λ_e_ values of **PDI-BI**. This indicates that the electron transfer rates of **PDI-BI-13**, **PDI-BI-14**, and **PDI-BI-16** are higher than that of **PDI-BI**. For –CH_3_ substituent molecules, the –CH_3_ group in different positions affects the λ_e_ values of **PDI-BI** slightly and decreases the λ_h_ values of **PDI-BI,** except the –CH_3_ group in 8-position, which can increase the the λ_h_ values of **PDI-BI**. This shows that the –CH_3_ substituent can improve the hole transport property of **PDI-BI**. The λ_e_ values of **PDI-BI-4**, **PDI-BI-13**, and **PDI-BI-14** are smaller than that of the typical electron transport material tris(8-hydroxyquinolinato) aluminium(III) (Alq3) (λ_e_ = 0.276 eV) [42], indicating that their electron transfer rates are higher than that of Alq3. The λ_h_ values of molecules **PDI-BI-1-26** are smaller than that of *N*,*N*′-diphenyl-*N*,*N*′-bis(3-methlphenyl)-(1,10-biphenyl)-4,40-diamine (TPD) (λ_h_ = 0.290 eV), which is a typical hole transport material [43]. This implies that their hole transfer rates are higher than that of TPD. Among these molecules, **PDI-BI-13** has the best electron transport property, and **PDI-BI-21** has the best hole transport property.

## 3. Materials and Methods

### Computational Methods

All the calculations were performed with the Gaussian 09 software [44]. Our previous work [31] suggested that the DFT method CAM-B3LYP with the 6-31G(d,p) basis set was reliable for optimization of PDI and its derivatives, and the TD-B3LYP/6-31+G(d,p) was reasonable for optical property simulation. Hence, the CAM-B3LYP/6-31G(d,p) method was employed to optimize all the geometry including neutral, cation, and anion **PDI-BI-1-26** molecules. The absorption spectra of **PDI-BI-1-26** molecules were predicted by the B3LYP/6-31+G(d,p) method. The PBE1PBE/6-31G(d) method was used to optimize the geometry of molecules **X1** and **X2** [32], and the HOMO and LUMO energies of molecules **X1** and **X2** were calculated at the CAM-B3LYP/6-31G(d,p) level on the basis of the single point energy. The B3LYP/6-31G(d,p) functional was successful in calculating the charge transport parameters [45]. Thus, we calculated the single point energy at the B3LYP/6-31G(d,p) level. The necessary parameters, such as single point energies of neutral, cation, and anion molecules in the ground state (S0), were recomputed for calculating the electronic properties of the molecules. The reorganization energy (λ) was predicted on the basis of the single point energy at the B3LYP/6-31G(d,p) level optimised neutral, cationic, and anionic geometries. Herein, the environmental relaxation and changes were ignored, and the reorganization energy of the isolated active organic π conjugated systems was the internal reorganization energy. As a result, Equations (1) and (2) can be used for calculating the values of electron reorganization energy (λ_e_) and hole reorganisation energy (λ_h_) [46]:

λ_e_ = [*E*_0_^−^ − *E*_−_] + [*E*_−_^0^ − *E*_0_] (1)

λ_h_ = [*E*_0_^+^ − *E*_+_] + [*E*_+_^0^ − *E*_0_] (2)

*E*_0_^+^ and *E*_0_^−^ are the cation and anion single point energies obtained by the optimized structure of the neutral molecule. *E*_+_ and *E*_−_ are the cation and anion single point energies calculated on the basis of the optimized structures of cation and anion molecules. *E*_+_^0^ and *E*_−_^0^ are the neutral single point energies obtained via the optimized structures of cation and anion molecules. *E*_0_ is the neutral single point energy calculated by the optimized structure of the neutral molecule at S0.

## 4. Conclusions

In the present work, we report a theoretical investigation predicting the substitution effects on optical and electronic properties for **PDI-BI**. The calculated results show that the substituents slightly affect the distribution patterns of FMOs for **PDI-BI**. The –CN and –NO_2_ groups in different substituent positions can decrease the *E*_HOMO_, *E*_LOMO_, and *E*_g_ of **PDI-BI**. The –CH_3_ group in different substituent positions affects the *E*_HOMO_, *E*_LOMO_, and *E*_g_ of **PDI-BI** slightly. The –CN group in different positions could increase the λ_abs-max_ and λ_abs-min_ values of **PDI-BI**, respectively, and the –CN group in the 5, 6, 7, or 8-position can increase the *R* values of **PDI-BI**. The –NO_2_ group in 2-position could produce a larger increase in λ_abs-max_ and λ_abs-min_ values, and the –NO_2_ group in 3-position could produce a larger decrease of the *R* value of **PDI-BI**. The –CH_3_ groups in different positions slightly affect the λ_abs-max_, λ_abs-min_, and *R* values of **PDI-BI**. Among these molecules, **PDI-BI-14** has the largest λ_abs-max_ value and **PDI-BI-6** has the largest *R* value. The –CN group in different positions can decrease the λ_e_ values and increase the λ_h_ values of **PDI-BI**. In the –NO_2_ substituent molecules, the substituent groups can increase the λ_e_ and λ_h_ values of **PDI-BI**. The –CH_3_ group in different positions slightly affects the λ_e_ values, and decreases the λ_h_ values of **PDI-BI**. **PDI-BI-13** and **PDI-BI-21** have the best electron and hole transport properties, respectively. On the basis of these results, we suggest that **PDI-BI-13**, **PDI-BI-14**, **PDI-BI-15**, **PDI-BI-16**, and **PDI-BI-17** are suitable acceptors for **X1** and **X2**. This study should be helpful in further theoretical investigations on such systems and also in the experimental study of solar cell acceptor materials.

## Figures and Tables

**Table 1:** Chemical structure of **PDI-BI** derivatives (Rn are –H except for mentioned in the Table). 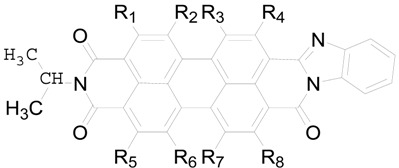

Molecules	R-Groups	Molecules	R-Groups
**PDI-BI-1**	R_1_ = –CN	**PDI-BI-14**	R_3_ = –CN R_6_ = –CN
**PDI-BI-2**	R_2_ = –CN	**PDI-BI-15**	R_3_ = –CN R_6_ = –NO_2_
**PDI-BI-3**	R_3_ = –CN	**PDI-BI-16**	R_4_ = –CN R_5_ = –NO_2_
**PDI-BI-4**	R_4_ = –CN	**PDI-BI-17**	R_4_ = –NO_2_ R_5_ = –NO_2_
**PDI-BI-5**	R_5_ = –CN	**PDI-BI-18**	R_3_ = –NO_2_ R_6_ = –NO_2_
**PDI-BI-6**	R_6_ = –CN	**PDI-BI-19**	R_1_ = –CH_3_
**PDI-BI-7**	R_7_ = –CN	**PDI-BI-20**	R_2_ = –CH_3_
**PDI-BI-8**	R_8_ = –CN	**PDI-BI-21**	R_3_ = –CH_3_
**PDI-BI-9**	R_1_ = –NO_2_	**PDI-BI-22**	R_4_ = –CH_3_
**PDI-BI-10**	R_2_ = –NO_2_	**PDI-BI-23**	R_5_ = –CH_3_
**PDI-BI-11**	R_3_ = –NO_2_	**PDI-BI-24**	R_6_ = –CH_3_
**PDI-BI-12**	R_4_ = –NO_2_	**PDI-BI-25**	R_7_ = –CH_3_
**PDI-BI-13**	R_4_ = –CN R_5_ = –CN	**PDI-BI-26**	R_8_ = –CH_3_

**Table 2:** The predicted *E*_HOMO_, *E*_LOMO_, *E*_g_, λ_abs-max_, λ_abs-min_, and *R* values of **PDI-BI** and its derivatives at the TD-B3LYP/6-31+G(d,p)//CAM-B3LYP/6-31G(d) Level.

	*E*_HOMO_	*E*_LOMO_	*E*_g_	λ_abs-max_	λ_abs-min_	*R*
**PDI-BI**	−6.88	−2.44	4.44	570.40	269.50	300.90
**PDI-BI-1**	−7.14	−2.74	4.40	581.24	282.68	298.56
**PDI-BI-2**	−7.14	−2.75	4.39	580.46	281.54	298.92
**PDI-BI-3**	−7.16	−2.75	4.41	572.27	281.43	290.84
**PDI-BI-4**	−7.15	−2.72	4.43	569.68	278.77	290.91
**PDI-BI-5**	−7.13	−2.74	4.39	588.55	278.64	309.91
**PDI-BI-6**	−7.12	−2.76	4.36	595.24	280.40	314.84
**PDI-BI-7**	−7.14	−2.76	4.38	590.79	281.67	309.12
**PDI-BI-8**	−7.14	−2.75	4.39	592.00	280.06	311.94
**PDI-BI-9**	−7.17	−2.74	4.43	574.08	297.82	276.26
**PDI-BI-10**	−7.12	−2.71	4.41	584.98	300.92	284.06
**PDI-BI-11**	−7.19	−2.70	4.49	557.27	298.59	258.68
**PDI-BI-12**	−7.18	−2.72	4.46	564.87	298.98	265.89
**PDI-BI-13**	−7.40	−3.00	4.40	584.00	289.49	294.51
**PDI-BI-14**	−7.39	−3.05	4.34	596.19	291.72	304.47
**PDI-BI-15**	−7.40	−3.00	4.40	585.19	305.80	279.39
**PDI-BI-16**	−7.43	−3.01	4.42	581.03	307.99	273.04
**PDI-BI-17**	−7.45	−3.02	4.43	582.91	311.98	270.93
**PDI-BI-18**	−7.43	−2.95	4.48	567.95	343.18	224.77
**PDI-BI-19**	−6.86	−2.39	4.47	564.68	270.26	294.42
**PDI-BI-20**	−6.83	−2.37	4.46	559.84	271.85	287.99
**PDI-BI-21**	−6.84	−2.37	4.47	557.89	271.51	286.38
**PDI-BI-22**	−6.85	−2.39	4.46	564.76	269.00	295.76
**PDI-BI-23**	−6.85	−2.39	4.46	567.34	270.18	297.16
**PDI-BI-24**	−6.82	−2.37	4.45	564.37	272.33	292.04
**PDI-BI-25**	−6.82	−2.37	4.45	562.73	272.19	290.54
**PDI-BI-26**	−6.84	−2.39	4.45	571.83	270.17	301.66

**Table 3:** Calculated λ_e_ and λ_h_ (eV) values of **PDI-BI** and its derivatives.

	λ_e_	λ_h_
**PDI-BI**	0.298	0.210
**PDI-BI-1**	0.278	0.222
**PDI-BI-2**	0.277	0.215
**PDI-BI-3**	0.278	0.221
**PDI-BI-4**	0.272	0.222
**PDI-BI-5**	0.282	0.226
**PDI-BI-6**	0.285	0.224
**PDI-BI-7**	0.286	0.230
**PDI-BI-8**	0.278	0.232
**PDI-BI-9**	0.296	0.225
**PDI-BI-10**	0.360	0.236
**PDI-BI-11**	0.290	0.222
**PDI-BI-12**	0.320	0.234
**PDI-BI-13**	0.265	0.240
**PDI-BI-14**	0.266	0.240
**PDI-BI-15**	0.343	0.249
**PDI-BI-16**	0.279	0.245
**PDI-BI-17**	0.312	0.264
**PDI-BI-18**	0.476	0.250
**PDI-BI-19**	0.297	0.201
**PDI-BI-20**	0.296	0.201
**PDI-BI-21**	0.296	0.195
**PDI-BI-22**	0.298	0.200
**PDI-BI-23**	0.299	0.205
**PDI-BI-24**	0.298	0.206
**PDI-BI-25**	0.298	0.200
**PDI-BI-26**	0.300	0.213

## Abbreviations

PDI	Perylene diimide
OSCs	Organic solar cells
PCEs	Power conversion efficiencies
FMOs	Frontier molecular orbital energies
HOMO	Highest occupied molecular orbital
LUMO	Lowest unoccupied molecular orbital
DFT	Density function theory
